# Research progress of the netrins and their receptors in cancer

**DOI:** 10.1111/jcmm.18241

**Published:** 2024-03-28

**Authors:** Xing Gao, Jiazhou Ye, Xi Huang, Shilin Huang, Wenfeng Luo, Dandan Zeng, Shizhou Li, Minchao Tang, Rongyun Mai, Yongqiang Li, Yan Lin, Rong Liang

**Affiliations:** ^1^ Department of Digestive Oncology Guangxi Medical University Cancer Hospital Nanning Guangxi China; ^2^ Department of Hepatobiliary Surgery Guangxi Medical University Cancer Hospital Nanning Guangxi China

**Keywords:** cancer, membrane, netrins, receptor, secreted

## Abstract

Netrins, a family of secreted and membrane‐associated proteins, can regulate axonal guidance, morphogenesis, angiogenesis, cell migration, cell survival, and tumorigenesis. Four secreted netrins (netrin 1, 3, 4 and 5) and two glycosylphosphatidylinositols‐anchored membrane proteins, netrin‐G1 and G2, have been identified in mammals. Netrins and their receptors can serve as a biomarker and molecular therapeutic target for pathological differentiation, diagnosis and prognosis of malignant cancers. We review here the potential roles of the netrins family and their receptors in cancer.

## 1 INTRODUCTION

Cytokines, complement components, growth factors, peptide hormones and immunoglobulins are actively released from cells to exert crucial biological functions. These secreted proteins not only contribute to the modulation of energy metabolism and immune responses but also assume pivotal functions in diverse aspects such as tumour cell growth, proliferation, invasion, angiogenesis and resistance to therapeutic agents.^1^

The netrins genes, which encode a family of secreted and membrane‐associated proteins that function as chemotropic guidance signals for directing cell and axon migration throughout nervous system development, were initially found in 1994 by Serafini et al.^2^ In mammals, four secreted netrins, netrin‐1 (NTN1), netrin‐3 (NTN3), netrin‐4 (NTN4) and netrin‐5 (NTN5) and 2 glycosylphosphatidylinositols (GPI)‐anchored membrane proteins, netrin‐G1 (NTNG1) and netrin‐G2 (NTNG2), have been reported. Netrin‐2 (NTN2) is homologous to NTN3, and all other netrins, but it is a distinct branch of the netrin family and has been identified in flies, nematodes and vertebrates.

## 2 THE STRUCTURE OF NETRINS

Netrins are also members of the laminin‐related protein superfamily, and their N‐terminal domains and three epidermal growth factor (EGF)‐like repeats are homologous domains to the N‐terminal domains VI and V of laminins (Table 1). NTN 1, 2 and 3 are structurally similar to the short arms of laminin γ chains. In addition, they also have a laminin VI domain and three EGF‐like repetitions (V‐1, V‐2 and V‐3) that are comparable to laminin V domains, as well as a positively charged C‐terminal structural domain (NTR/C345C).^3^ Whereas, the N‐terminal end of NTN4 is homologous to the laminin‐β1 chain, and it contains a laminin VI domain, a laminin V domain (domains V‐1 to V‐3) and a C345C domain. NTN5 has three EGF motifs in the laminin V domain and the C345C domain, but lacks the N‐terminal laminin VI domain, unlike other netrin‐secreted proteins.^4^

**TABLE 1:** The basic information of the human Netrins family.

Gene	Types	Location	Amino acids	Size (kDa)
NTN1	Secreted netrins	17p13.1	604	67.7
NTN3	Secreted netrins	16p13.3	580	61
NTN4	Secreted netrins	12q22	628	70
NTN5	Secreted netrins	19q13.33	489	53.2
NTNG1	Membrane‐associated proteins	1p13.3	539	60.5
NTNG2	Membrane‐associated proteins	9q34.13	530	59.8

Abbreviations: NTN1, netrin‐1; NTN3, netrin‐3; NTN4, netrin‐4; NTN5, netrin‐5; NTNG1, netrin‐G1; NTNG2, netrin‐G2.

In addition, netrin G proteins also have the same architecture, which includes a laminin VI domain, three LE motifs from the laminin V domains and a hydrophobic GPI lipid anchor at the C‐terminus. Unlike the secreted netrins, the C‐terminal sequence (known as domain C′) of netrin‐G is hydrophobic and serves as a signal for GPI linkage to the coupling and increased netrin‐G located on the extracellular surface of the plasma membrane.^5^

## 3 NETRINS RECEPTORS FAMILY

**FIGURE 1:**
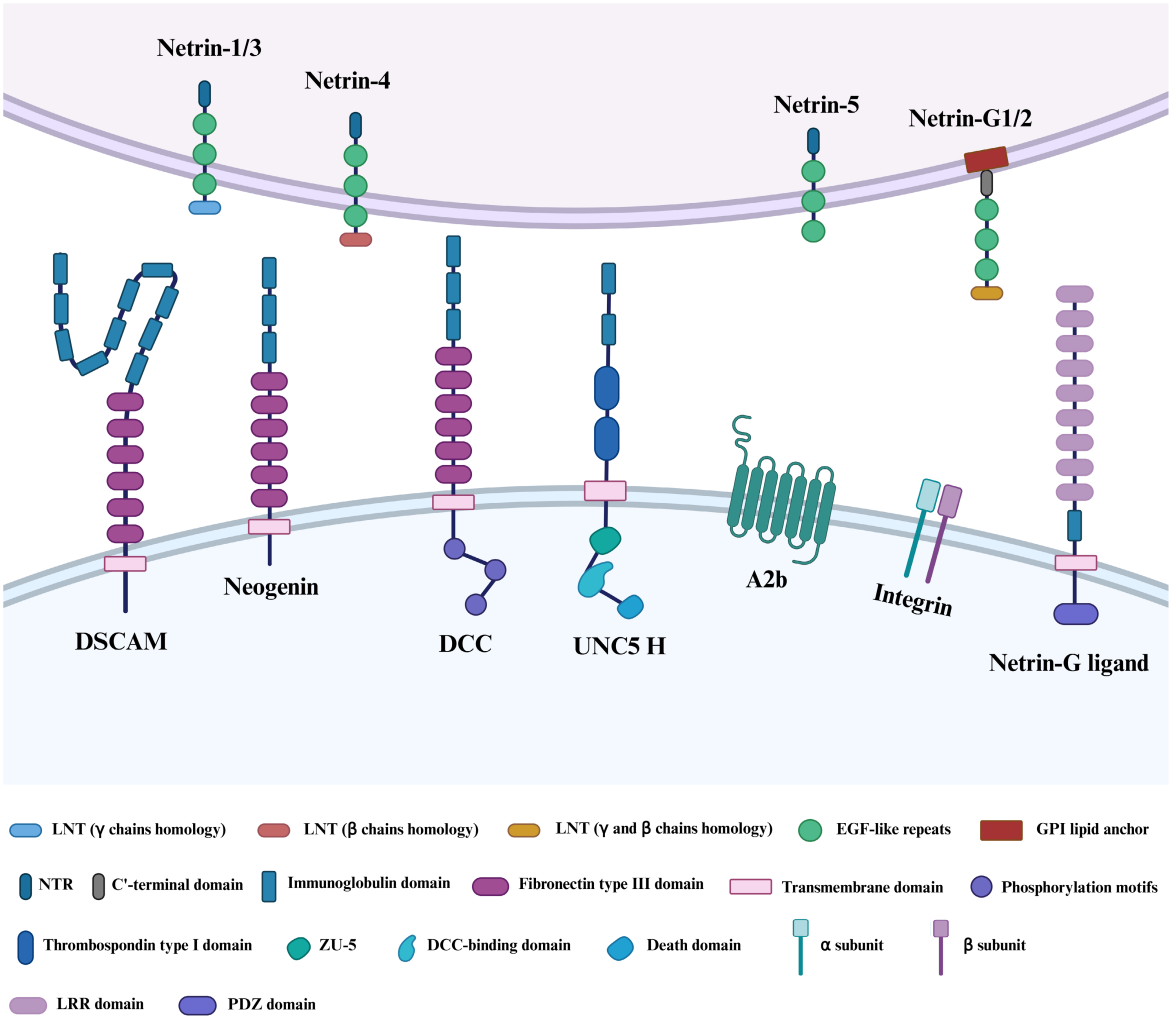
Structure of netrins and their receptors. DCC, deleted in colorectal cancer; DSCAM, Down syndrome cell adhesion molecule; GPI, glycosylphosphatidylinositol.

In mammals, the receptors that interact with secreted netrins include deleted in colorectal cancer (DCC), the analogous neogenin (NEO1) and the uncoordinated 5 homologue (UNC5H) family. Additionally, the receptors include the Down syndrome cell adhesion molecule (DSCAM) family, adenosine receptor A2b (A2b) and integrin.^6^ Netrin‐G membrane‐bound proteins do not appear to interact with these receptors, but they do regulate the neuron interactions via binding to netrin G ligands NGL‐1 and NGL‐2 on the transmembrane netrin G ligands NGL‐1 and NGL‐2 (also known as LRRC4C and LRRC4, respectively).^7^ Apart from A2b and integrin, the identified netrin receptors are predominantly single‐pass type I transmembrane proteins. These receptors belong to the Ig superfamily and exhibit a consistent structural pattern, encompassing an extracellular domain, a transmembrane region and an intracellular domain (Figure 1).

The first netrin receptors belong to the DCC subfamily of proteins, which was first identified in humans as a possible tumour suppressor and is known for high‐frequency loss in colorectal tumours.^8^ DCC is a 1447 amino acid protein encoded by the DCC gene located on the human chromosome 18q21.3. The DCC receptor is made up of four Ig‐like domains organized in a horseshoe arrangement outside the cell, followed by six fibronectin type III (FN III) domains, with evidence indicating that FNIII repeat numbers 4, 5 and 6 are the DCC domains that bind netrin.^9^ It is well known that upon netrins binding, homodimerization of DCC via the P3 motif is required to activate chemoattraction processes. Intracellularly, DCC does not encode any obvious catalytic domain, but it does contain three highly conserved sequences known as P1–P3 motifs, which play a crucial role in transmitting signals downstream upon the binding of netrins.^10^

NEO1, a homologue of the DCC receptor group, encodes a 1461 amino acid protein and shares about 50% amino acid identity with DCC. They have a similar secondary structure, consisting of an extracellular domain, a transmembrane domain and an intracellular domain.^11^ A novel regulatory role in signal transduction pathways is now emerging. Such as axon guidance, cell adhesion, differentiation, proliferation, migration, angiogenesis and tissue growth through interaction.^12^

UNC5H is a prominent member of the extensive netrin receptors family, featuring four members in humans: UNC5A, UNC5B, UNC5C and UNC5D, also known as UNC5H1–4 in rodents.^13^ In contrast to DCC, the UNC5 receptors exhibit a distinctive structural composition in their extracellular segment, containing two Ig domains along with two thrombospondin (TSP) domains. Notably, the Ig repeats play a pivotal role in facilitating the binding of netrins. Intriguingly, the intracellular domain of UNC5 receptors comprises three conserved domains: a ZU5 domain, a domain that interacts with DCC (DB domain) and a death domain (DD). A caspase‐3 cleavage site in the cytoplasmic DD region is involved in downstream signalling leading to apoptosis.^14^ In the absence of DCC, the presence of UNC5H is sufficient to turn attraction into short‐range chemorepellent responses to netrins independently. In the presence of DCC, DCC can form heterodimers with UNC5H by coupling of the P1 domain of DCC with the intracellular DB domain of UNC5H to facilitate long‐range chemoemetic responses.^15^

DSCAM is a large (>200 kDa) immunoglobulin superfamily neural cell adhesion protein that is found on human chromosome band 21q22.2 and plays a crucial role in Down syndrome.^16^ The structural configuration of the DSCAM protein is widely acknowledged to be conserved throughout bilaterians, containing 10 Ig domains and six FNIII repeats domains. Notably, the tenth Ig domain is positioned between FNIII 4 and FNIII 5. Furthermore, a TM transmembrane fragment and C‐terminal intracellular domain are also included.^17^ In the past, DSCAM was considered an orphan receptor without related ligands. Recent studies have proved that the Ig domains of DSCAM can bind to netrins and provide netrin‐dependent axon guidance throughout development. However, the signalling mechanisms engaged by netrins downstream of DSCAM remain unknown.

The A2b is one of four adenosine receptor subtypes that belong to the class A family of G protein‐coupled receptors (GPCRs). It regulates a variety of functions, including vascular tone, neurosecretion, tumour cell proliferation and metastasis.^18^, ^19^ Corset et al.^20^ found that DCC and A2b interact with the NTN1 to induce cAMP accumulation upon binding adenosine. It demonstrates that contact with the adenosine A2b receptor is required for netrin‐mediated axon extension and cAMP generation.

Integrins are cell adhesion receptors that are evolutionarily old and are made up of paired α‐ and β‐subunits. In vertebrates, there are 18 α‐subunits and 8 β‐subunits, which may assemble into 24 distinct integrins depending on their ligand‐binding properties or subunit composition.^21^ A recent publication shows that integrins α6β4 and α6β4 can interact with netrins to regulate the netrin‐mediated cell function and signalling, such as cell growth, proliferation, survival, migration and differentiation.^22^, ^23^ Moreover, integrins are large family of transmembrane proteins, not all of which are receptors for netrins.

NGLs are single‐pass transmembrane proteins from the leucine‐rich repeat (LRR) superfamily, consisting of an extracellular domain with nine LRR regions capped by N‐ and C‐terminal cysteine‐rich domains, followed by a C2‐type Ig domain.^24^ These are followed by a transmembrane domain and a cytoplasmic domain of about 100 amino acids in mammals, which includes the PDZ‐binding motif at the C‐terminus. Type I transmembrane proteins NGL‐1 and NGL‐2 have LRR and Ig domains in their extracellular domains.^25^ Fascinatingly, NGL‐3 (also known as LRRC4B), is the third member within the NGL family member found in mammals. Unlike its counterparts NGL‐1 and NGL‐2, NGL‐3 does not exhibit an affinity for binding either NTNG1 or NTNG2. Instead, it does interact with transmembrane receptor tyrosine phosphatases LAR (PTPRF) and protein tyrosine phosphatase δ (PTPδ). This interaction suggests that NGL‐3 is involved in the development and function of excitatory synapses.^26^

**FIGURE 2:**
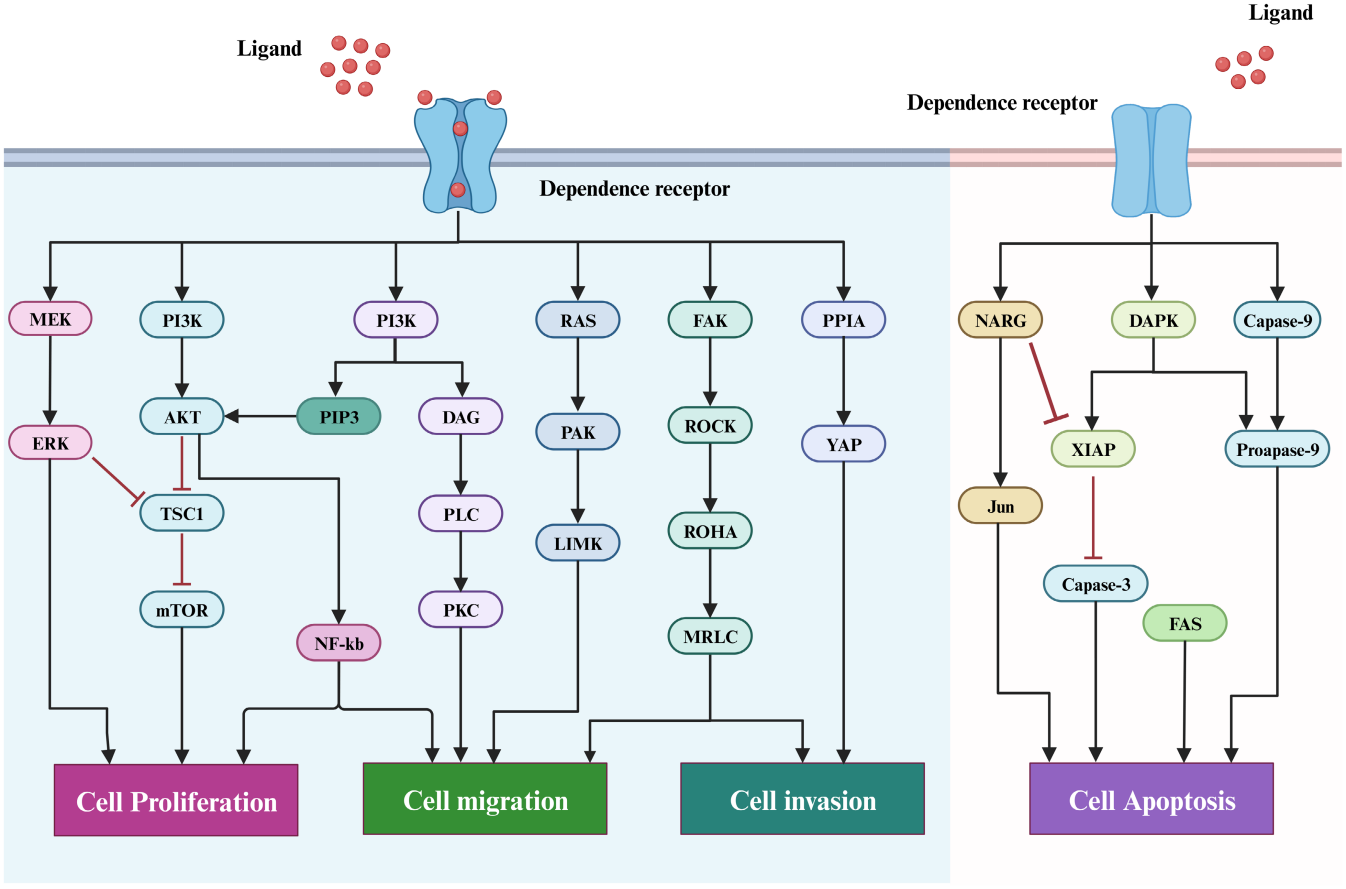
Dependence receptors have two properties: when the ligand is present, binding to the ligand promotes cell survival, migration or differentiation. When the ligand is absent, it will induce apoptosis. The function of this receptor depends on the survival rate of its ligand. Binding of netrins to its receptors leads to multiple classic associated signalling pathway activation such as RAS/GTP, MAPK/ERK, FAK/ROCK and PI3K/AKT signalling.

Apart from in the nerve cells, netrins receptors are also found in many other types of cells, such as tumour cells, vascular endothelial cells and epithelial cells, among others. In addition, the DCC, UNC5H, integrin, and NEO1 are all members of the dependence receptors (DRs) family, which are characterized by functional similarities and activate two distinct signalling pathways depending on whether or not ligand is present. These receptors form multimers in the presence of ligands, transmitting a variety of signals for cell survival, migration and differentiation, as well as participating in a variety of physiological and pathological processes. The receptor is monomeric in the absence of the ligand, resulting in an intracellular area that can be cleaved by cysteinase and the release of the C‐terminal fragment, which then activates the downstream proapoptotic pathway.^27^ As a result, the survival of cells with these receptors in the extracellular environment is dependent on the presence of ligands (Figure 2). Therefore, it is crucial for tumour therapy to understand the mechanisms and regulatory implications of netrins and its receptor interactions in tumour development.

## 4 NETRINS AND TUMOURS

Current studies have shown that netrins, as a potential oncogene or tumour suppressor gene in multiple human cancers, regulates tumor growth and are associated with disease diagnosis and prognosis. In addition, the underlying mechanisms of netrins are an intricate biological process, which is affected by many tumour factors and signal pathways.^28^ Therefore, this study reviews the recent studies on netrins and malignant tumours.

### 4.1 Neuroblastoma

Neuroblastoma (NB), which originates from neural crest cells that give rise to sympathetic nerves, is the most prevalent extracranial solid tumour in children. According to the International Neuroblastoma Risk (INSS) classification, which encompasses five stages in the case of NB, namely 1, 2, 3, 4 and 4S.^29^

Related experiments showed that NTN1 and NTN4 were highly expressed in NB cell lines and tumour tissues and were positively correlated with poor prognosis. In addition, low expression of NTN1 and NTN4 not only promoted NB cell apoptosis and reduced cell proliferation, migration and invasion in vitro, but it also inhibited NB tumour formation and metastasis in vivo, suggesting that they are oncogenic genes in NB.^30^, ^31^ Another study by Jiang et al.^29^ first found that the NB patients at stage 4 showed higher NTN3 levels, and NTN3 was associated with overall survival and poor prognosis. These results imply that netrins play an important role in NB cell survival and carcinogenesis.

In NB, the mode of action of netrins and their receptors is also a complex biological process, which is regulated by multiple tumour star molecules and signalling pathways. Mehlen et al.^30^ reduced the expression of NTN1 in NB cells and found that the UNC5H/DAPK signal pathway was triggered, resulting in an increase in cell death while inhibiting the dissemination and transfer of tumour cells in the nude mice. MYCN activation is a hallmark of advanced NB and is a primary oncogene driving this malignancy.^32^ A recent study confirmed that the NTN3 high‐expression group had excessive activation of MYCN‐regulated pathways. The ChIP‐sequencing analysis detected a notable enrichment of MYCN binding sites in the NTN3 promoter locus, which supports NTN3 as a target gene regulated by MYCN in NB.^29^ Consequently, netrins and their receptors might be promising prognostic and therapeutic targets for NB.

### 4.2 Glioblastoma

Glioblastoma (GBM) is the most malignant form of glioma, which is the most frequent primary tumour of the central nervous system.^33^

In glioma tissues, the expression of NTN1 was increased and positively correlated with tumour grade and malignancy. Notably, subsequent investigations^34^ NTN1 promoted tumour cell proliferation and tumour growth in a mouse xenograft model via inducing NF‐κB p65ser536 phosphorylation and increasing c‐Myc expression. Interestingly, the activation of NF‐κB by NTN1 is dependent on the UNC5A receptor, as UNC5A can trigger the NF‐κB p65ser536 phosphorylation and induce the upregulation of c‐Myc expression. These findings imply that NTN1, a promising therapeutic target for the treatment of GBM, may potentially activate the NF‐κB signalling pathway through UNC5A to promote glioma cell proliferation in GBM. Hu et al.^35^ investigated the expression levels of primary tumours from The Cancer Genome Atlas‐Glioblastoma Multiforme (TCGA‐GBM) repository and discovered that NTN4 expression was downregulated in GBM and was associated with poor patient survival. Subsequent in vitro studies showed that NTN4 influenced GBM cell proliferation and migration in a concentration‐dependent way. Furthermore, it was discovered through molecular mechanism research that NTN4 interact to integrin beta‐4 (ITGB4) to facilitate the phosphorylation of AKT and mTOR, while concurrently diminishing the phosphorylation of ERK1/2.^36^ This revealed that NTN4/ITGB4 stimulated the phosphorylation of the AKT‐mTOR signalling pathway, which in turn increased GBM cell proliferation.

Temozolomide (TMZ), an alkylating agent, is now the first‐line chemotherapeutic medication for GBM treatment. It induces GBM cell apoptosis and senescence by triggering DNA damage, so as to achieve the therapeutic effect of GBM. Li et al.^35^, ^36^ found that NTN4 can cooperate with ITGB4 to induce activation of the PI3K‐AKT‐mTOR signalling pathway, which increases phosphorylation levels of AKT and mTOR, thereby protecting cells from TMZ‐induced cellular senescence and enhancing the drug resistance of GBM cells to TMZ. After that, Li^37^ conducted the RNA‐sequencing data from NIH 934 human cell lines and discovered that NTN4 expression was highly correlated with EGFR, and that EGFR expression was also tightly linked to ITGA6 and ITGB4. NTN4/ITGB4 was shown to protect GBM cells from TMZ‐induced cellular senescence, and GBM patients with EGFR/NTN4 co‐expression had a poor prognosis. Additionally, NTN1 can also regulate the expression of c‐Myc by activating the two signal pathways NTN1/NEO1/FAK/ITGB4 β1 and NTN1/UNC5B/NEO1/Gsk3α/β, so as to participate in the control of the GBM neonatal vascular formation process, thereby promoting GBM cell proliferation, invasion and migration.^38^ Overall, the complex function of netrins in GBM cells is mediated by different receptors and multiple signalling pathways, thus elucidating its key molecular mechanisms that may provide effective targets for targeted therapy of GBM.

### 4.3 Breast cancer

As one of the most frequent malignant tumours in women globally, breast cancer (BC) poses a major threat to human survival and well‐being.^39^

Fitamant et al.^40^, ^41^ conducted experiments showing that the plasma levels of NTN1 protein levels were significantly higher in BC patients than that in control patients, and NTN1 was significantly elevated in N + M0 tumours than in N0 tumours (median, 1.8 vs. 0.5; *p* = 0.007). Moreover, Wischhusen et al.^42^ used ultrasound molecular imaging (USMI) to show that USMI signalling could reflect NTN1 mRNA and protein expression levels in a breast tumour model. This imaging method was non‐invasive and safe, and it could provide the NTN1 expression status in near real‐time. Therefore, NTN1 has the potential to provide a new idea for BC diagnosis and gene therapy, which could be an auxiliary diagnostic approach for the stratification and diagnosis of BC patients.

Consistent with this, studies^43^ have reported that NTN4 overexpression enhances the phosphorylation of intracellular signalling components AKT, ERK in vitro, induces the growth of lymphatic and blood vessels, and leads to enhanced metastasis in human and mouse mammary carcinoma cancer cells in vivo. These data suggest that NTN4 acted as an oncogene in BC. In contrast, studies by Xu et al.^44^ showed that the levels of NTN4 mRNA and protein were decreased in breast cancer tissues compared to paracancerous tissues, which was consistent with the oncomine database. In addition, NTN4 overexpression inhibited breast cancer cells proliferation, migration, invasion and tumour formation, as well as downregulation in N‐cadherin and vimentin expression.^45^ These results demonstrate that NTN4 also plays a role as a suppressor gene in BC. Therefore, NTN4 was involved in cancer, but the exact role of NTN4 appeared to be dependent on the cancer type.

In BC cells, netrins receptors also regulate tumour growth and metastasis. Studies have found that α6β4 was typically expressed in the breast epithelium and elevated in invasive breast cancer, and it could increase the ability of BC cells to grow and invade by activating the PI3K/AKT signalling pathway.^46^ Consistently, UNC5C inhibits breast cancer metastasis by downregulating MMP9 expression through PI3K/AKT, ERK and p38 MAPK signalling pathways.^47^, ^48^ In addition, UNC5C also interacts with integrin α6β4 to prevent breast cancer cell proliferation and metastasis.^23^ Therefore, the NTN1/α6β4/UNC5C signalling pathway is critical for regulating tumour growth and metastasis.

Death‐associated protein kinase1 (DAPK1), a serine‐threonine kinase responsible for UNC5H‐induced apoptosis, has been found to be downregulated in a variety of malignancies. Mechanistic analysis^49^ has shown that decitabine (5‐aza‐2′‐deoxycytidine, DAC) reduces DNA methylation of the DAPK1 promoter region, leading to an elevation in DAPK1 expression, suggesting that inhibiting DNA methylation could be a viable therapy option. Concordantly, Grandin et al.^50^ demonstrated that decitabine inhibited DNA methylation, effectively rejuvenating the expression of NTN1 and DAPK1 in NTN1‐low cancer cells. In addition, the combination of decitabine and NTN1 accentuated tumour cell death and effectively restraint of tumour development in animal models. Therefore, combining DNA methylation inhibitors with NTN1 may be an effective anticancer therapy.

### 4.4 Gastric cancer

Gastric cancer (GC) ranks as the fourth most prevalent cancer. Due to the lack of early diagnostic markers, patients are often diagnosed at an advanced stage.^51^

Kai et al.^52^ found that NTN1 was upregulated in GC tissues and cell lines compared to adjacent normal ones, and it was linked to neural invasion (NI), lymph node metastasis, depth of invasion and distant metastasis in GC patients. NTN1 knockdown suppressed cell migration abilities, invasion abilities and the migratory potential of GC cells along the nerve in GC cells.^53^ Furthermore, NTN1 concentrations in advanced GC were higher than in a healthy control group, and NTN1 concentrations reduced after treatment. Similarly, Li et al.^54^ discovered the high expression of NTN4 was negatively correlated with patient survival time but positively correlated with the severity of pathological grading in tumour tissues and serum samples from GC patients. Therefore, netrins have potential values as tumour markers in clinical applications and may provide new directions for GC research and treatment.

In addition, the expression levels of netrins receptors vary significantly during different stages of GC.^55^, ^56^ In GC at an early stage, the abnormal methylation of both the DCC and UNC5C genes led to increased gene transcription levels, while in advanced gastric carcinoma, this phenomenon gradually disappeared, and NTN1 levels were found to be further elevated. In contrast to DCC, NEO1 appeared to have varying expression and function in GC. Studies^12^, ^54^ have found that NEO1 was upregulated in GC patients and cells and NTN4 overexpression mediated NEO1‐induced decreased phosphorylation of Stat3, ERK, AKT and p38 and enhanced GC cell proliferation and invasion, suggesting that NTN4 may be involved in GC development through multiple oncogenic pathways (JAK/STAT, PI3K/AKT and ERK/MAPK). Chemotherapy is the major treatment option for GC patients with unresectable tumours, with cisplatin (DDP) serving as the first‐line medication.^57^ Moreover, Qu et al.^58^ discovered that overexpression of NEO1 reduced GC cell sensitivity to cisplatin while increasing cell survival, motility and adhesion, but silencing of NEO1 had the reverse effect. According to the findings mentioned above, netrins are a positive regulator of malignant carcinogenesis and metastasis in GC. In addition, the key mechanism of netrins regulating cell proliferation and invasion via their receptor NEO1 also provides a new entry point for the treatment of GC.

### 4.5 Pancreatic cancer

Pancreatic cancer is a highly malignant disease of the digestive tract, with pancreatic ductal adenocarcinoma (PDAC) being the most prevalent pathological subtype.^59^

Studies^60^ have shown that NTN1 and its gene product were substantially overexpressed in PDAC tissue samples, and patients with high NTN1 expression had a very poor prognosis. In pancreatic cancer cells PANC1 and CFPAC1, Dumartin et al.^61^, ^62^ overexpressed NTN1 and found that MDM2 expression levels increased, Cyclin D1 expression declined, which then induced enhanced cellular invasiveness in vivo and in vitro, and provided adhesion substrates for tumour cells. These results suggest that NTN1 can serve as a tumour marker for PAAD and activate the UNC5B/FAK and Mdm2 signalling pathways, thereby inhibiting the growth of PDAC.

The pan‐cancer landscape of netrin family^1^ revealed that NTN4 was highly expressed and NTNG2 was lowly expressed in the blood of PAAD patients. Furthermore, survival analysis revealed that NTN4 higher expression and NTNG2 lower expression were negatively connected with patient prognosis. Subsequently, Francescone et al.^63^ conducted experiments and revealed that NTNG1 was upregulated in PDAC tissues and that high NTNG1 expression was negatively correlated with patient survival. Moreover, the results demonstrated that NTNG1 overexpression promotes PDAC tumorigenesis in nude mice. Further function and mechanism analysis proved that NTNG1 functions were mediated through the AKT and p38 pathways in PDAC. According to the research mentioned above, Netrins may be a potential new treatment target and have great application value in clinical practice for PAAD.

### 4.6 Colorectal cancer

Colorectal cancer (CRC) is the third most common malignancy and the fourth leading cause of cancer death.^64^

Recent evidence suggests that netrins play an important role in colorectal carcinogenesis. Li et al.^65^ reported that NTN1 had higher expression in CRC tissues and serums than in normal, and might be a tumour marker of CRC. Eveno et al.^66^ further found that high expression of NTN4 inhibited the primary tumour growth and recurrence of colorectal xenografts in nude mice, while also inhibiting the tumour number and liver metastasis volume of the orthotropic liver metastasis model. Therefore, NTN4 may be a valuable biomarker and a potential therapeutic target in advanced CRC. Subsequently, Shonan et al.^67^ used bioinformatics analysis and discovered that NTNG1 exhibited hypermethylation mutations in CRC patients, with the mutation frequency being highest at the EGF‐like 3 locations in the NTNG1 laminin EGF domains. In addition, individuals with the NTNG1 mutation had a higher risk of recurrence and a worse prognosis than those without the mutation. Therefore, netrins may aid in the selection of adjuvant therapy as well as the customization of surveillance regimens.

The recent discovery that netrins and its receptors interact to regulate CRC cell proliferation and apoptosis. Sung et al.^68^ discovered that UNC5C was commonly hypermethylated in colorectal tissues and associated with bigger polyp size, older age, histology, and mutant BRAF, implying that UNC5C may relate to the malignant progression of CRC. Their further research demonstrated that the timing of molecular mutations in UNC5C and DCC was not random, with UNC5C inactivation occurring in early tumour lesions and DCC locus changes forming through progressive accumulation. Both NTN1 and A2B‐R induce the aggressive phenotype in DCC‐deficient human CRC cells through the Rho‐Rho kinase axis. Furthermore, ectopic NTN1 expression accelerated the growth of human colon tumour xenografts.^69^ As a transcription factor, Yes‐associated protein (YAP) activates the hippo signalling pathway and plays an important role in cancer development and progression. For further function and mechanism analysis of netrins and their receptors in CRC, Qi et al.^70^ proved that NTN1 vied its transmembrane receptors, DCC and UNC5H, stimulating phosphatase 1A to dephosphorylate YAP and inhibiting the ubiquitination and degradation. It enhanced YAP accumulation and increased YAP levels in the nucleus, thereby promoting CRC cells proliferation and migration. Their research showed that NTN1 exerted oncogenic activity through the activation of the YAP signalling pathway, providing a mechanism to couple extracellular signals with nuclear YAP oncogenes. These findings indicated that DCC was a metastasis suppressor gene that targeted both pro‐invasive and survival pathways, whereas NTN1 was a strong invasion and tumour growth‐promoting agent.

### 4.7 Lung cancer

Lung cancer (LC) is the second most prevalent type of cancer and is divided into two subtypes, small cell lung cancer (SCLC) and non‐small‐cell lung cancer (NSCLC), of which NSCLC is the most common histological subtype.^71^

Bourgeois et al.^72^ detected clinical samples from 92 patients with NSCLC (45 adenocarcinomas and 47 squamous cell carcinomas) and found that upregulated NTN1 expression was detected both in adenocarcinomas and squamous cell cancers, and NTN1 expression was more frequent and more intense in adenocarcinomas than in squamous cell carcinomas. It is worth noting that NTN1 overexpression promoted NSCLC cell invasion, migration and angiogenesis mimicry (VM), while NTN1 knockdown reversely dampened the metastatic potential. Mechanism analysis confirmed that NTN1 could induce EMT by activating the PI3K/AKT signalling pathway, suggesting that NTN1 may become a new promising target for NSCLC.^73^ Overall, the current findings demonstrate a distinct role for netrins in the development of LC under hypoxic conditions and provide further evidence that netrins may be used as therapeutic targets, offering promise as a potential target for LC treatment drugs. Similarly, another study^28^ showed that NTN1 interference promotes EMT via the PI3K/AKT pathway in the hypoxic microenvironment of NSCLC, suggesting that plasma NTN1 could be used as a diagnostic and prognostic biomarker in these patients. Moreover, Jiang et al.^29^ also detected that NTN3 was significantly upregulated in SCLC tissues compared with normal tissues and that knocking down NTN3 markedly inhibited the tumour formation ability of nude mice, suggesting that NTN3 is involved in the occurrence of SCLC. In addition, Hao et al.^1^ applied bioinformatics and biological data mining and found that NTNG1 was differentially expressed in adenocarcinoma and squamous patients, with low expression of NTNG1 in lung adenocarcinoma and high expression of NTNG1 in lung squamous cell carcinoma all being associated with poor prognosis of patients, indicating that NTNG1 is also involved in the occurrence and development of LC. Collectively, this research identified a potential molecular therapeutic target for NSCLC.

### 4.8 Ovarian cancer

Ovarian cancer (OC) is the second leading cause of mortality among women with gynaecological cancers worldwide.^74^

Previous research has discovered that NTN1 is overexpressed in the vast majority of malignant ovarian tumours but not in benign tumours. Moreover, the correlation between high NTN1 expression and tumour stage and grade suggests that NTN1 may perform an oncogene role in OC.^75^ Another study by Li et al.^76^ found the expression levels of SOX6 and NTN1 were opposite in PA‐1 and SW626 cells. Moreover, NTN1 overexpression significantly inhibited the expression of SOX6, reversing the effects of SOX6 on cell proliferation and invasion in vitro and tumour xenograft growth and vascularity in vivo. Thus, NTN1 might be a crucial regulator of ovarian cancer progression and a promising biomarker.

High‐grade serous ovarian cancer (HGSOC) is the most prevalent subtype of ovarian cancer, and its poor prognosis is largely attributed to a late‐stage diagnosis with metastases already present.^77^ Dormant or minimal residual disease that is not clinically detectable is a common source of relapse, particularly in a metastatic setting.^78^ HGSOC disseminates as growth‐arrested cellular aggregates, indicating that they are ideal for searching for survival dependencies in dormant cells. Perampalam et al.^79^ found that overexpression of NTN1 and NTN3 induces elevated survival in dormant HGSOC culture conditions and disease spread in a mouse xenograft model of dissemination. Moreover, the dependence of ERK phosphorylation on UNC5H and NEO1 receptors demonstrates it is lower when receptors are deleted. Mechanistic analysis showed that Netrins signal through a heterodimer or multi‐receptor complex containing both an UNC5 and NEO1 component that provides stimulation for MEK‐ERK axis in the absence of mitogenic signals in dormant culture conditions. Overall, Netrins and MEK are feasible new candidates for dormant HGSOC disease.

Currently, cisplatin is the first‐line chemotherapy treatment for recurrent platinum‐sensitive ovarian cancer, functioning either through DNA damage or activation of the endoplasmic reticulum (ER) stress pathway. However, DDP treatments perform poorly against ovarian cancer that is resistant to platinum.^80^ Fang et al.^81^ found that ovarian cancer patients with low NTNG1 expression had a longer platinum‐free interval (PFI) and progression‐free survival (PFS) and were more sensitive to cisplatin. The knockdown of NTNG1 led to the upregulation of p‐AXL, p‐AKT and RAD5, as well as an increase in cisplatin sensitivity, but the opposite was observed when NTNG1 was overexpressed in SKOV3/DDP cells. In vivo investigations demonstrated that NTNG1 influenced the therapeutic efficacy of cisplatin: overexpression of NTNG1 decreased therapeutic efficacy, whereas downregulation improved anticancer activity, which was consistent with in vitro findings. The following mechanism was proposed as a result of these findings: NTNG1 directly binds GAS6/AXL, regulating AXL and AKT phosphorylation, upregulating RAD51 expression, enhancing DSB repair and finally leading to cisplatin resistance. As a result, NTNG1 was identified as a potential therapeutic target for ovarian cancer, and blocking NTNG1 might be a viable method for overcoming cisplatin resistance.

### 4.9 Bladder cancer

Bladder cancer (BC) is the fourth most prevalent malignant disease of the urinary system, and it is one of the most common malignant diseases in males worldwide.^82^

Liu et al.^83^ found that NTN1 expression was lower in bladder cancer cells than in normal bladder cell lines. Its expression was positively associated with histological grade, T stage, metastasis and poor prognosis in bladder cancer tissues. Furthermore, receiver operating characteristic curve (ROC) analysis supported the muscle‐invasion diagnostic significance of urine NTN1 in bladder cancer patients, with an area under the curve of 0.758, 96% sensitivity and 67% specificity. NTN1 gene expression was also found to be a positive predictive sign for local tumour recurrence in Cox regression models.^84^ There is evidence that NTN1 may serve as a diagnostic marker for breast cancer.

UNC5B showed lower expression in bladder cancer tissues compared with adjacent normal tissues. Higher levels of UNC5B expression were associated with a poor prognosis and a short overall survival time in people with bladder cancer.^83^ UNC5B overexpression in 5637 BC cells decreased cell multiplication and migration, as well as produced cell cycle arrest in the G2/M phase, with alterations in the expression of cell cycle‐associated proteins, indicating that UNC5B may suppress bladder cancer cells' metastatic tendencies.^85^ The mechanism is that PKC inhibitor PMA treatment boosted BC cells' proliferative and invasive activity, while PKC agonist caliphostin C therapy inhibited it.^83^ Furthermore, strong PKC activity, high NTN1 expression and low UNC5B expression can improve bladder cancer cells cisplatin tolerance. Conversely, low PKC activity, low NTN1 expression and high UNC5B expression can increase the susceptibility of bladder cancer cells to chemical therapeutics. Additionally, PKCα high activity and NTN1 high expression concomitantly lead to diminished UNC5B expression while simultaneously enhancing the NTN1/UNC5B combination.^86^ Consequently, PKCα and NTN1/UNC5B work together to regulate the effects of cisplatin on bladder cancer cells through a positive feedback regulatory loop. Similarly, Liu et al.^87^ demonstrated that NTN1 acted as a direct target of miR‐214. A negative connection was also detected between NTN1 and miR214 expression in bladder cancer tissues. In addition, cisplatin may increase NTN1 protein expression in bladder cancer cells, whereas miR214 mimics may partially prevent this effect. NTN1 plasmid transfection reduced cisplatin‐induced apoptosis, enhanced AKT phosphorylation and decreased caspase3 and PARP cleavage. Plasmid‐transfected miR‐214 mimic cells recovered NTN1. In conclusion, the current work indicates that miR‐214 reduces chemoresistance by targeting NTN1 in bladder cancer cell lines. In conclusion, the current work suggested that miR‐214 reduces chemoresistance by targeting NTN1 in bladder cancer cell lines.

### 4.10 Hepatocellular carcinoma

As the most common form of liver cancer, hepatocellular Carcinoma (HCC) is the sixth most common cancer and the fourth most common cause of cancer‐related mortality worldwide.^88^

Compared to their normal counterparts, upregulated NTN1 expression was detected in HCC tissue specimens and cell lines. A high level of NTN1 was linked to MCV and lymph node metastasis. In addition, NTN1, released from hypoxic HCC cells, can induce EMT‐promoted invasion. In contrast, targeted reduction of endogenous NTN1 using shRNA dramatically decreased the invasion and metastasis of HCC cells.^89^ These findings demonstrated that NTN1 increases downstream signalling pathways in hypoxic HCC cells, resulting in EMT activation and the generation of several inflammatory mediators that promote cancer invasion. In addition, Wen et al.^90^ constructed a pathway network that comprise the miRNA target genes associated with HBV‐associated HCC. Among them, the most weight value of NTN1 in signalling pathway and regulation of frizzled proteins (FZD) by ubiquitination, at 0.228. The liver is a major model of inflammation‐associated cancer development, leading to HCC. The proinflammatory effect of NTN1 on chemokines and Ly6C^+^ macrophages was also discovered by Romain et al.,^91^ suggesting that hepatic inflammation triggers the generation of proinflammatory NTN1 by exclusively activating translation. Therefore, NTN1 would be beneficial to provide valuable insights into the diagnosis and therapeutic strategies of HBV‐related HCC.

Blood vessel epicardial substance (BVES) was first discovered as a possible cell adhesion molecule with a crucial function in maintaining epithelial integrity and controlling cell migration. Recent studies^92^ have revealed that NTN1 is highly expressed and BVES is downregulated in HCCC tissues and cell lines compared to adjacent normal ones. Surprisingly, the levels of NTN1 and BVES had a negative connection. Importantly, upregulating BVES expression reduced NTN1‐enhanced motility and invasion, while silencing BVES expression recovered the NTN1 knockdown cells' metastatic behaviour. NTN1 enhances HCC cell migration and invasion in part by modulating BVES expression through the PI3K/AKT signalling pathway, according to molecular mechanism research. In conclusion, the NTN1/BVES pathway might be a key target for HCC therapeutics as well as a source of potential biomarkers for HCC identification and prognosis. Similarly, Yan et al.^89^ observed a similar role of NTN1 that was discovered in HCC, prompting a new mechanistic hypothesis. They hypothesized that NTN1 activated downstream signalling pathways in hypoxic HCC cells, causing EMT and the generation of numerous inflammatory mediators, which promote cancer invasion. Moreover, our research team^93^ has found that NTNG1 was highly expressed in HCC cell lines and tumour tissues and was positively associated with poorer OS rates. NTNG1 overexpression in HCC cells significantly increased their proliferation, invasion, migration, and EMT, while inhibiting apoptosis. Collectively, our results are the first to identify NTNG1 functions as an oncogene in HCC and may serve as a potential novel therapeutic target. In conclusion, these investigations suggested that netrins might be used as a viable therapeutic target for HCC therapy (Table 2).

**TABLE 2:** Murine studies on netrins and their receptors in the tumour.

Cancer type	Expression	Upstream	Receptors	Downstream	Outcome
Neuroblastoma	NTN1 (↑)	–	DCC, NEO1, UNC5H	UNC5H/DAPK, FAK, NF‐Κb, RhoA	Cell proliferation, migration and apoptosis
	NTN3 (↑)	MYCN	–	–	Cell apoptosis
	NTN4 (↑)	–	NEO1	–	Cell proliferation, migration and apoptosis
Colorectal cancer	NTN1 (↑)	–	DCC, UNC5H	NF‐κB, cAMP/PKA, Rho/ROK, Hippo	Tumour growth, cell proliferation, migration and apoptosis
	NTN4 (↓)	–	NEO1	VEGF/FGF	Angiogenesis, cell migration and tumour recurrence and metastasis
	NTNG1	–	–	–	Tumour recurrence and Patient prognosis
Breast cancer	NTN1 (↑)	–	DCC, UNC5H, α6β4	DAPK, NF‐κB, EGFR/EGR, PI3K/AKT	Cell proliferation, migration and invasion
	NTN4 (↑↓)	–	α6β1	Rho GTPases, Src/FAK	Cell migration, invasion and angiogenesis
Gastric cancer	NTN1 (↑)	–	DCC, UNC5H, NEO1	PI3K‐AKT, ERK/MAPK, Hippo	Cell proliferation, migration and invasion
	NTN4 (↑)	–	NEO1	Jak/Stat, PI3K‐AKT, ERK/MAPK	Cell proliferation, migration and invasion
Pancreatic cancer	NTN1 (↑↓)	–	DCC, UNC5H, NEO, ITGB4	p53/Mdm2, MEK‐ERK, FAK	Cell proliferation
	NTN4 (↑)	–	–	–	Patient prognosis
	NTNG1 (↑)	–	–	AKT/4E‐BP1, p38/FRA1	Cell growth and patient prognosis
	NTNG1 (↓)	–	–	–	Patient prognosis
Glioblastoma	NTN1 (↑)	–	UNC5H	Notch, NF‐κB	Cell migration, invasion and angiogenesis
	NTN4 (↑)	–	ITGB4	PI3K‐AKT‐mTOR, EGF/EGFR	Cellular senescence and patient prognosis
Lung cancer	NTN1 (↑)	ITGB4	DCC	PI3K/AKT, ERK	Cell proliferation, migration, invasion and angiogenesis
		IGF2BP1			
	NTN3 (↑)	–	–	–	Tumour growth
	NTNG1 (↑)	–	–	–	Patient survival
Ovarian cancer	NTN1 (↑)	SOX6	–	–	Cell proliferation and migration
		–	UNC5H, NEO1	MEK‐ERK	Tumour growth
	NTN3 (↑)	–	UNC5H, NEO1	MEK‐ERK	Tumour growth
Bladder cancer	NTN1 (↑)	–	UNC5H	–	Cell proliferation and migration
Liver cancer	NTN1 (↑)	–	DCC, UNC5H	PI3K/AKT, NF‐κB, Hippo	Cell migration and angiogenesis
	NTNG1 (↑)	–	–	–	Cell proliferation, migration and invasion
Haematological malignancies	NTN1 (↑)	–	DCC	–	Cell apoptosis and tumour growth
		–	UNC5B	FAK‐MAPK	Cell apoptosis
		–	–	Egr1	Cell quiescence and self‐renewal
Melanoma	NTN1 (↑)	–	DCC, UNC5H	–	Cell migration, invasion and angiogenesis
Cervical cancer	NTN4 (↓)	miR‐196a	–	–	Cell proliferation and migration

Abbreviations: DAPK1, death‐associated protein kinase1; DCC, deleted in colorectal cancer; ITGB4, integrin beta‐4; NEO1, neogenin; NTN1, netrin‐1; NTN3, netrin‐3; NTN4, netrin‐4; NTNG1, netrin‐G1; UNC5H, uncoordinated 5 homologue.

### 4.11 Haematological malignancies

Haematological malignancies are classified into three types: leukaemia, lymphoma and multiple myeloma (MM).^94^

B‐cell lymphoblastic leukaemia (B‐ALL) and acute myeloid leukaemia (AML) are the most common childhood malignancies. Huang et al.^95^, ^96^ observed that NTN1 was highly expressed in the blood of B‐ALL and AML patients. Mechanism analysis confirmed that NTN1 induces B‐ALL and AML cell anti‐apoptotic effects by interacting with the receptor UNC5B and activating the FAK‐MAPK signalling pathway. In human diffuse large B‐cell lymphoma (DLBCL) and mantle cell lymphoma (MCL) biopsies tissues, upregulation of the NTN1/DCC expression ratio tipped the scales towards loss of DCC‐induced apoptosis. Additionally, treatment with the NTN1‐interfering antibody slowed down tumour growth and increased survival in lymphoma mouse xenograft models.^97^ After that, studies by David et al.^98^ showed that there was a clear decrease in MM cell viability when treated with NP137 in combination with cytotoxic agents in mice with established myeloma tumours.

Studies have found that the different cells of the immune system originate from the haematopoietic stem cells (HSCs).^99^ In NTN1^*β‐geo/β‐geo*^ mouse models, increased activation and progressive loss of HSC numbers as well as self‐renewal potential were observed after global deletion of NTN1, mimicking the NEO1‐mutant phenotype. In addition, overexpression of NTN1 enhanced HSC quiescence in vivo and in vitro. Mechanistically, NTN1 induces Egr1 expression and maintains the dynamic balance of quiescence and self‐renewal of HSCs in a NEO1‐dependent manner.^100^

Based on these findings, netrins may be a prospective therapeutic target for haematological malignancy patients.

### 4.12 Other cancers

In addition to the aforementioned cancers, high expression of NTN1 and low expression of UNC5B were found in tumour cells of bladder cancer, prostate cancer, melanoma and renal cell carcinoma.^101^, ^102^, ^103^ NTN1 knockdown induced increased expression of UNC5B and significantly weakened cell migration and invasion abilities in melanoma.^103^ In addition, under hypoxic conditions, NTN1 knockdown inhibited the activation of YAP and the ability of cell migration and invasion, preventing the occurrence of EMT.^104^ However, another found that NTN4 was downregulated in cervical cancer tissue, and low expression of NTN4 promoted the capacity of cell proliferation and migration in cervical cancer.^105^, ^106^ In summary, the role of netrins in regulating the malignant growth of tumour cells deserves further study.

## 5 THE MUTATIONS OF NETRINS AND THEIR RECEPTORS IN CANCERS

Recent cancer studies have also found that the many netrins and their receptor variants of proteins play vital roles in the development of some cancers.

The basement membrane (BM) is a specialized extracellular matrix mainly composed of laminin, and the vascular BM (vBM) along the endothelium stabilizes newly formed vessels and promotes their maturation.^107^ Reuten^108^ found that NTN4 forms a high‐affinity complex with laminin γ1, blocks laminin polymerization and then disrupts laminin networks and BMs, whereas its respective laminin‐binding mutants (NTN4‐ΔC and NTN4‐ΔC^E195A,R199A^) cannot. Moreover, upon NTN4 treatment, vascular endothelial cell lines were inhibited and vessel‐like network stability was reduced, whereas the laminin‐binding mutant showed no effect. These findings propose that the anti‐angiogenic property of NTN4 in vivo is directly related to its impact on BM rearrangement. In addition, NTNG1 exhibited hypermethylation mutations in CRC patients, with the mutation frequency being highest at the EGF‐like 3 locations in the NTNG1 laminin EGF domains.^67^ Individuals with the NTNG1 mutation had a higher risk of recurrence and a worse prognosis than those without the mutation. Further analysis of the mutations revealed that mutations in netrin genes in each genetic ancestry group were mainly concentrated in the laminin N‐terminal and laminin EGF domains.^1^

The timing of molecular mutations in UNC5C and DCC was not random, with UNC5C inactivation occurring in early tumour lesions and DCC locus changes forming through progressive accumulation.^68^ Nakamura et al.^109^ identified previously unreported somatic mutations of UNC5C (9.9%) via next‐generation sequencing. They also revealed a significant positive association between mutations in DNA repair genes and improved clinical outcomes in bladder cancer patients. Another study showed that UNC5A mutations had poorer PFS in head and neck squamous cell carcinoma and prostate adenocarcinoma.^102^ Moreover, Mehlen et al.^110^ have also reported that while the wild‐type UNC5C receptor is proapoptotic in the absence of its ligand, the UNC5C (A628K) mutant is no longer effective in triggering apoptosis.

## 6 THE VITAL ROLES AND MECHANISM OF NETRINS IN CANCER DIAGNOSIS AND THERAPY

Specifically, netrins and their receptors have been shown to occur in multiple human cancers. In preclinical models mimicking these diseases, interference between netrins and their receptors was sufficient to trigger cancer cell death.^72^, ^111^ Based on these findings, a monoclonal antibody (mAb) neutralizing NTN1 and blocking the NTN1‐UNC5B interaction, dubbed NP137, was preclinically developed and tested in various clinical trials.^112^

Blanpain et al. reported that NTN1 is elevated in mouse models of endometrial adenocarcinoma (EC),^113^ skin squamous cell carcinoma (SCC)^114^ and myeloma tumours^98^ and that treated intraperitoneally with NP137 is effective in reducing tumour progression. Another study by Kryza et al.^115^ discovered that NTN1 is actually poorly diffusible and only after bound to the extracellular matrix, then it could be released and diffusible in the microenvironment during development. The researchers then used this property to radiolabelled NP137 with lutetium‐177 (^177^Lu) and discovered that a single systemic injection of NP137‐^177^Lu with high specificity provides important antitumor effects and prolonged mouse survival in tumour cell‐engrafted and genetically engineered mouse models. Further research revealed, in addition to EC cell death induction, that administration of NP137 prevented the progression of cancer cells towards a late EMT state. Furthermore, a combination of NP137 and carboplatin‐paclitaxel outperformed carboplatin‐paclitaxel alone in the EC mouse model.^113^ Therefore, the multifaceted use of NP137 is not only for diagnostic but also therapeutic purposes.

Notably, netrin‐targeted therapy appeared to have an effect on immune cells. Pten f/f EC mice treated with NP137 had an increase in CD8^+^ cells.^113^ NTN1 knockdown in tumour cells decreased the immunosuppressive activity of myeloid‐derived suppressive cells (MDSCs) and restored antitumor immunity in MC38 tumour xenograft mice, according to Xia et al.^116^ The molecular mechanism investigation revealed that NTN1 significantly enhanced the immunosuppressive function of MDSCs through A2BR on MDSCs, thus promoting the development of tumours. In addition, Ducarouge et al.^117^ discovered that NTN1 interference limits tumour resistance to immune checkpoint inhibitors and provided evidence linking this enhanced anti‐tumour efficacy to a decreased recruitment of a subtype of MDSCs called polymorphonuclear (PMN)‐MDSCs.

In a Phase I trial comprising 14 patients with advanced endometrial adenocarcinoma (EC), Cassier et al.^113^ observed eight stable illnesses (57.1%) and one objective response (partial response, 7.1%). In addition, they also found a decrease in the proportion of tumour cells co‐expressing pancytokeratin and vimentin by IHC staining in patients with the EC described above. Single‐cell analysis also showed that there was a statistically significant decrease in cancer cells and in the EMT score across the whole tumour compartment in EC patients. Immunological changes observed from single‐cell RNA‐seq analysis demonstrated a clear switch from monocytes in C1D1 to dendritic cells in C3D1 interacting with cancer cells, indicating NP137 treatment is associated with more efficient antigen‐presenting cells (APCs). Together, this supports the view that NP137, possibly by impacting EMT, enhances the tumour immune response.

Scholars are presently broadening their scope to investigate the feasibility of employing the medication to treat a multitude of additional malignancies, such as hepatocellular carcinoma (NCT05546879, Phase I), pancreatic ductal adenocarcinoma (NCT05546853, Phase I), advanced and metastatic solid tumours (NCT05605496, Phase II), cervix carcinoma (NCT04652076, Phase I/II) and acute myeloid leukaemia refractory (NCT06150040, Phase I/II). For now, all the clinical trials of NP137 showed an excellent safety profile and basically no toxicity related to target inhibition.

## 7 CONCLUSION AND FUTURE PERSPECTIVES

In this review, we summarized the diverse activities of netrins and their receptors in several cell types under physiological and pathological settings. In particular, increasing attention has been focused on the contradictory roles of netrins and their receptors in numerous human malignancies. The pro‐ and anti‐tumour effects are influenced by a variety of factors, including cell origins, receptor types, relative concentration and relative expression. Consequently, there is a need for additional research to elucidate the precise roles that netrins play in the context of various diseases.

## AUTHOR CONTRIBUTIONS

**Xing Gao:** Conceptualization (equal); visualization (equal); writing – original draft (equal). **Jiazhou Ye:** Conceptualization (equal); funding acquisition (equal); writing – review and editing (equal). **Xi Huang:** Conceptualization (equal); visualization (equal); writing – original draft (equal). **Shilin Huang:** Conceptualization (equal). **Wenfeng Luo:** Investigation (equal). **Dandan Zeng:** Investigation (equal). **Shizhou Li:** Investigation (equal). **Minchao Tang:** Investigation (equal). **Rongyun Mai:** Investigation (equal). **Yongqiang Li:** Investigation (equal). **Yan Lin:** Conceptualization (equal); funding acquisition (equal); writing – review and editing (equal). **Rong Liang:** Conceptualization (equal); funding acquisition (equal).

## FUNDING INFORMATION

This work was supported by the National Natural Science Foundation of China (no. 82060427, 82103297); Guangxi Medical and health key discipline construction project; Guangxi Scholarship Fund of Guangxi Education Department; Nanning Qingxiu District Science and Technology Project (no. 2021007, 2021010, 2021012); Advanced Innovation Teams and Xinghu Scholars Program of Guangxi Medical University; Guangxi Medical University Outstanding Young Talents Training Program and Youth Program of Scientific Research Foundation of Guangxi Medical University Cancer Hospital (no. 2023‐7).

## CONFLICT OF INTEREST STATEMENT

The authors declare that they have no competing interests.

## Data Availability

The authors declare that they have no competing interests.
